# Interventions That Failed: Factors Associated with the Continuation of Bullying After a Targeted Intervention

**DOI:** 10.1007/s42380-023-00169-7

**Published:** 2023-04-10

**Authors:** Eerika Johander, Tiina Turunen, Claire F. Garandeau, Christina Salmivalli

**Affiliations:** 1 INVEST Flagship Research Center/Department of Psychology and Speech-Language Pathology, University of Turku, 20014 Turku, Finland; 2 Shandong Normal University, Jinan, China

**Keywords:** Bullying, Victimization, Intervention, School

## Abstract

We examined how often teachers’ targeted interventions fail in stopping bullying and to what extent this varies between schools vs. between students involved. In addition, we investigated which student-level factors were associated with intervention failure. Data were collected annually in 2011–2016 via online questionnaires and included responses from students in 2107 Finnish primary and secondary schools implementing the KiVa antibullying program. During the years of the study, 27% of the 57,835 students who were victims in the cases of bullying addressed by adults reported no improvement in their situation. Among the 44,918 bullying perpetrators who were targeted by an intervention, 21% said they did not bully less as a result. Intervention failures were mostly due to differences between individuals: only 3–12% of the total variance in continued victimization and bullying was due to between-school differences. According to two-level logistic regression results, victim-perceived failure was more likely when the victimized student was in higher grades, had been victimized more frequently and, for a longer time, had been victimized also online, had bullied others, and had fewer friends in the class. Bully-perceived failure was more likely when the bullying student was in higher grades, bullied more frequently, and was victimized. Finally, the bullying students’ antibullying attitudes and their perception of teacher’s and parents’ antibullying attitudes were negatively associated with failure of the intervention.

## Introduction

Studies on bullying prevention and intervention have mostly focused on the efficacy of whole-school programs (Gaffney et al., 2019b; Ttofi & Farrington, 2011)—often emphasizing their success, as they lead to lower average levels of bullying perpetration and victimization than “practice as usual.” Although researchers have started to tease apart the contributions of different program components from the overall effects (Gaffney et al., 2021a; Hensums et al., 2022), little is known about the effects of *targeted interventions*—by which we refer to intervening in cases of bullying as they emerge (e.g., discussions with the students involved). However, there is evidence that as many as 20–50% of adult actions aiming to stop bullying (repeated aggressive attacks against a relatively powerless peer) fail—even in the context of whole-school programs (Garandeau et al., 2014b; Johander et al., 2021; Salmivalli, 2023; van der Ploeg et al., 2016); in other words, bullying perpetration or victimization often continues after the intervention. The present study examines intervention failure in the context of the Finnish antibullying program KiVa (Kärnä et al., 2011b) from both victimized students’ and bullying perpetrators’ perspectives and student-level characteristics associated with the failure.

### Failure of Targeted Interventions: Prevalence

Most estimates regarding the effects of targeted interventions are based on retrospective student reports of what happened when they were bullied by peers at school. The findings suggest that teacher interventions fail in putting an end to ongoing bullying in about half of the cases and sometimes even make the situation worse (Davis & Nixon, 2011; Fekkes et al., 2005; Rigby, 2014; Rigby & Barnes, 2002; Smith & Shu, 2000). It seems, however, that many studies focus on immediate responses of teachers when witnessing bullying incidents, rather than intervening in the victimized student’s situation as a whole (see Salmivalli, 2023). In a recent study asking students to recall bullying incidents they had either witnessed or been involved in as perpetrators or victims, 23.6% of students reported that, after teacher intervention, bullying did not stop in the short term and 34.6% said it did not stop in the long term (Wachs et al., 2019). In another study, students who reported being victimized and had sought help from a teacher were asked to indicate what happened to the bullying over the following few weeks (Rigby, 2020). Bullying had stopped or decreased in 67% of the cases and had stayed the same or gotten worse in 33% of the cases.

In a study examining the short-term effectiveness of targeted interventions conducted as part of the KiVa antibullying program in Finland, victimized students were asked in a follow-up meeting about 2 weeks after the intervention whether the bullying had stopped (Garandeau et al., 2014b). They reported that the bullying had stopped or decreased in 97.7% of the cases and the situation had remained the same or gotten worse in 2.4% of the cases. The long-term effectiveness of the targeted interventions was examined in a study conducted after the nationwide roll-out of the same program, using reports from victimized students collected annually across 6 years via online questionnaires (Johander et al., 2021). At the end of each school year, students who reported that they had been victimized and their situation had been addressed by the adults at school were asked whether the interventions had an impact on their situation. Students from 1221 primary and secondary schools responded. According to these (retrospective) reports, the bullying had stopped or decreased in 74% of the cases, and the situation had not changed or the bullying had increased in 26% of the cases. The interventions were less successful in secondary schools (grades 7–9) than in primary schools (grades 4–6), when teachers (according to their own reports) had used methods other than the program-recommended evidence-based ones and when no systematic follow-up discussions had been organized to ensure that the bullying had stopped. In these studies, the two program-recommended intervention methods (i.e., confronting vs. non-confronting approach; Garandeau et al., 2014b; Johander et al., 2021) were found to be equally effective. However, the interventions were less effective when the schools had used their own adaptation or when they could not specify which approach they had used (Johander et al., 2021).

### Factors Associated with Intervention Failure

Intervention failures from victimized students’ and bullying perpetrators’ perspectives are linked (if the bullying behavior does not stop, the targeted student’s victimization experiences are not likely to stop either)—but this is not to say that they never diverge. Although many of the potential factors associated with intervention failure are the same for both outcomes, some characteristics or perceptions predicting failure might be specific to victimized youth, others to the perpetrators. Most studies have relied on the victim’s perspective only (asking victimized students whether peers stopped bullying them), ignoring the perspective of the bully/ies. Indeed, we could locate only one study (Garandeau et al., 2016) where bullying perpetrators were asked whether they intended to stop bullying after being exposed to a targeted intervention.

The *age* of the students involved appears to play a role in how successful adult interventions are at stopping bullying and therefore victimization. Previous research has shown that both whole-school antibullying programs (Hensums et al., 2022; Yeager et al., 2015) and targeted interventions specifically (Johander et al., 2021) tend to be less effective in adolescence than in childhood. In addition, the *intensity* of the victimization or bullying (how often it happens, how long it has been taking place) might make interventions less likely to succeed. More frequent victimization might indicate that the victim is targeted by more than one perpetrator and thus make the situation harder to tackle, as group dynamics are involved and several individuals must be confronted. Indeed, frequency of being victimized by a group has been found to be negatively related to intervention success (Rigby, 2020). Also, the likelihood that victimization continues after a targeted intervention is higher when it has lasted longer (Garandeau et al., 2014b). In such cases, it is possible that other attempts to put an end to the bullying by victims themselves or by peers have already taken place and failed. Frequent bullying behavior, on the other hand, might be an indication of other co-occurring problems (e.g., aggressive-impulsive behavior and retaliatory attitudes) of the perpetrators that also need to be addressed in order to solve the problem (O’Brennan et al., 2009). Bullying may even have become habitual behavior, thus elicited with minimal cognitive effort, control, or intention (Gardner et al., 2019).

Whether the forms of bullying matter for intervention success is debatable. Anecdotally, online bullying is often mentioned as especially difficult to prevent or intervene against, as adults may not be aware that it has happened. However, research shows that it often co-occurs with face-to-face bullying (Cosma et al., 2020; Salmivalli et al., 2013) and can be addressed by school actions (Williford et al., 2013)—although perhaps not as effectively as other forms (Gaffney et al., 2019a, b). In online victimization, a single incident can be shared with a large audience (e.g., by forwarding a compromising picture to multiple people; Smith & Slonje, 2010) and the perpetrator can easily remain anonymous (Patchin & Hinduja, 2006). Indeed, instant messaging programs, pseudonyms in chat rooms, temporary accounts in social networking services, and other online venues can make it hard for adults to determine the perpetrator’s identity. In addition, electronic devices allow the perpetrators to contact the victim at any time and almost in any place (Patchin & Hinduja, 2006; Smith & Slonje, 2010), and victimization that occurs outside of school grounds and school hours might be harder to handle. Also, school personnel might not know how to address online bullying. In a study examining the capacity of Australian primary and secondary school personnel to address covert bullying, 50.8% of them felt not at all skilled or poorly skilled and only 8.2% felt very skilled to address online bullying (Barnes et al., 2012).

Specific to bullying perpetrators, there are some cognitions that are likely to contribute to behavioral change after an intervention. According to the theory of planned behavior (Ajzen, 1991), both individual attitudes and perceptions of important others’ attitudes (so-called subjective norms) matter. Thus, behavior change is more likely when the individual’s own attitudes are in line with the hoped-for behavior, but also when they feel that important others have attitudes supportive of change. Indeed, increases in students’ antibullying attitudes and increases in students’ perception of how disapproving of bullying their teacher is were found to predict reductions in bullying (Saarento et al., 2015). The attitudes of the bullying perpetrators’ parents are also likely to play a role in whether attempts to intervene in bullying will be successful. Studies that have examined the role of parental characteristics (such as acceptance of violence and positive attitudes for bullying and victimization) have found them to be related to bullying (for a review, see Nocentini et al., 2019). Parents’ normative beliefs about victimization have been associated with elevated levels of aggression (Troop-Gordon & Gerardy, 2012), and their use of physical discipline was found to predict elevated bullying behavior (Espelage et al., 2000). In contrast, spending time with adults who suggest non-violent strategies to manage conflicts has been associated with a reduced likelihood of engaging in bullying behavior (Espelage et al., 2000).

Some youth are *both* victimized by others and bully others themselves (e.g., van Dijk et al., 2017; Yang & Salmivalli, 2013). Addressing the victimization faced by these “bully-victims” might be challenging, as peers (and even adults) may feel that their negative treatment is justified due to their own aggressive behavior (Yang & Salmivalli, 2013). Bully/victims are often the most rejected students (Juvonen et al., 2003; Veenstra et al., 2005), which also means that intervening successfully to end their victimization might not be easy. On the other hand, addressing the bullying behavior of these youth can also be difficult as they may feel entitled to bully others due to their own plight as victims.

Besides individual characteristics, contextual factors might also affect the (non-) effectiveness of targeted interventions. There is good indication that the presence (or absence) of support from classmates might play an important role. Victimized children, especially those chronically victimized, tend to be lonely and lack social support (Acquah et al., 2016; Hawker & Boulton, 2000; Herráiz & Gutiérrez, 2016; Romera et al., 2021; Sheppard et al., 2019), whereas having at least one friend can protect against victimization (Hodges et al., 1999). Thus, having few or no friends might make it more likely that victimization continues even after an intervention.

### Current Study

Even when teachers do intervene in cases of bullying, their actions fail to put an end to bullying in a relatively high number of cases. However, few studies have tried to understand why this happens. This study addresses important gaps in the literature by examining how often targeted interventions by adults fail to stop bullying and which factors are associated with such failure.

First, using data from more than 2000 Finnish schools followed over a period of 6 years, we examine the prevalence of intervention failure, that is, how often victimization and bullying continue after adults’ targeted interventions. We use reports from both victimized students (whether their victimization stopped) and those who bullied others (whether they changed their behavior after the intervention). Second, we investigate the extent to which the varying outcomes of interventions are due to differences between individual students or differences between schools. Third, we identify factors that predict intervention failure at the individual level by examining the effects of grade level, frequency of victimization, frequency of victimized students’ own bullying behavior, presence of online victimization, and whether the victimized students have friends in the classroom on intervention failure from the victimized students’ perspective. Also, we examine the effects of grade level, frequency of bullying, frequency of bullying perpetrators’ own victimization, perpetrators’ antibullying attitudes, and their perceptions of the attitudes of their teachers and parents, on intervention failure from the bullying perpetrators’ perspective. We hypothesize that interventions are more likely to fail when the victimized student is in higher grades, has been victimized more frequently and, for a longer time, has been victimized online, has bullied others, and has fewer friends in the classroom. Furthermore, we hypothesize that intervention failures are more likely when the bullying student is in higher grades, has also been victimized, and has bullied others more frequently. We also hypothesize that the bullying students’ antibullying attitudes and their perceptions of teachers’ and parents’ antibullying attitudes are negatively associated with the intervention failure.

## Method

### Sample and Procedure

Data for the present study came from Finnish schools that were implementing the KiVa antibullying program (see description of the program in Kärnä et al. 2011b) between 2009 and 2016 and where students responded at least once to the annual online questionnaire (at the end of each school year) since in 2011. On average, schools had been implementing the program for 3.5 years, ranging from 0 (less than one academic year) to 7 years. The present study uses data from grades 4–9 because the questions related to targeted interventions (or “indicated actions”) were not asked from younger students (grades 1–3) who had a much shorter survey to fill in. A total of 838,695 students from 2107 schools responded to the questionnaire at least once between 2011 and 2016. This represents 77% of Finnish comprehensive schools (*n* = 2719; Official Statistics of Finland, 2022). Students responded to the surveys anonymously during regular school hours, using school-specific passwords to log in. The final sample consisted of data from 2032 schools in which at least some students reported being summoned to a discussion with adults at schools because they had either been victimized or had bullied others. The schools were from all around Finland: 1352 were primary schools (grades 1–6), 296 were secondary schools (grades 7–9), and 394 were combined (grades 1–9) schools.

Among the 2032 schools, 1901 provided reports from students who reported having being summoned to a discussion either because they had been victimized (*n* = 57,611) or had bullied others (*n* = 44,832). In 89 schools, reports were obtained only from victimized students (*n* = 224), and in 42 schools, data included only reports from students who had been bullying others (*n* = 86). Thus, 57,835 students in total reported being summoned to a discussion with adults at school because they had been victimized, and 44,918 reported being summoned to such a discussion because they had bullied others (that is 6.9% and 5.4% of the respondents).

### Measures

#### Victim-Perceived Intervention Failure

Students who reported that they had been victimized during the current school year and that the bullying they had experienced was addressed by adults at school were asked whether the intervention had an effect on their situation. The response options to the question “Did the adult intervention affect your situation?” were the following: (1) the situation did not change at all, I was still bullied, (2) since then I was bullied less or the bullying stopped completely, and (3) since then I was bullied more. For the analyses, responses one and three were compounded into one category “did not change at all/increased” and a dummy-coded variable (0 = decreased/stopped, 1 = did not change at all/increased) was created, the latter value representing intervention failure.

#### Bully-Perceived Intervention Failure

Students who reported that they had been bullying others and that their bullying behavior was addressed by an adult at school were asked whether the intervention had an effect on their behavior. Response options to the question “Did the adult intervention affect your behavior?” were the following: (1) the situation did not change at all, I continued bullying, (2) since then I bullied less or stopped bullying completely, and (3) I bullied more after that. For the analyses, responses one and three were compounded into one category “did not change at all/increased” and a dummy-coded variable (0 = decreased/stopped, 1 = did not change at all/increased) was created, the latter value again representing intervention failure.

#### Grade Level

Students were asked to indicate which grade (4–9) they were in.

#### Frequency of Victimization and Bullying

Self-reported frequency of victimization and bullying were measured using the global items from the revised Olweus’s Bully/Victim Questionnaire (Olweus, 1996). Responses to the questions “How often have you been bullied at school in the last couple of months?” and “How often have you bullied others at school in the last couple of months?” were given on a 5-point scale (0 = not at all, 1 = only once or twice, 2 = two or three times a month, 3 = about once a week, and 4 = several times a week).

#### Duration of Victimization

Students who reported that they had been bullied two or three times a month or more often during the last couple of months were asked to indicate how long the bullying had been going on. Responses to the question “How long have you been bullied?” were given on a 5-point scale (0 = a week or two, 1 = 1 month, 2 = about 6 months, 3 = 1 year, and 4 = many years).

#### Presence of Online Victimization

Students were asked to indicate whether they had been bullied online. Responses to the question “Have you been bullied through Internet during the past few months?” were given on a 5-point scale (0 = not at all, 1 = only once or twice, 2 = two or three times a month, 3 = about once a week, and 4 = several times a week). As we were interested in whether victimization included online forms (rather than their frequency), responses from 1 to 4 were compounded into one category of a dummy-coded variable (0 = no online victimization, 1 = presence of online victimization).

#### Friends in Classroom

Students were asked to indicate whether they had friends in their classroom. Responses to the statements “I have friends in my class” and “I have good friends in my class” were given in a 5-point scale (0 = I disagree completely, 4 = I agree completely). Before the analysis, scores of the two items were averaged. The reliability coefficient McDonald’s omega (see Hayes & Coutts, 2020) for these two questions was satisfactory (Ω = 0.85).

#### Antibullying Attitudes

Antibullying attitudes were measured with six items based on provictim scale (Rigby & Slee, 1991). Responses to items such as “It is okay to call some kids nasty names” (reverse coded) and “I feel bad seeing a child bullied” were given on a 5-point scale (0 = I disagree completely, 4 = I agree completely). Before the analysis, three negatively keyed items were reversely coded, and the mean score for antibullying attitudes was calculated (Ω = 0.76).

#### Perception of Teacher’s Antibullying Attitudes

Perception of teacher’s antibullying attitudes was assessed by asking “In your opinion, what does your teacher think about bullying?”. The responses were given on a 5-point scale (0 = my teacher thinks bullying is a good thing, 1 = my teacher does not care whether students are being bullied or not, 2 = I do not know, 3 = my teacher thinks that bullying is bad, 4 = my teacher thinks that bullying is absolutely wrong).

#### Perception of Parents’ Antibullying Attitudes

Perception of parents’ antibullying attitudes was assessed by asking “What do your parents (or guardians) think about bullying?”. Again, responses were given on a 5-point scale: (0 = they think bullying is a good thing, 1 = they do not care whether students are being bullied or not, 2 = I do not know, 3 = they think bullying is bad, 4 = they think bullying is absolutely wrong).

#### Control Variables

Control variables used in the analyses were (self-reported) gender of the student (0 = girl; 1 = boy) and the number of years the school had implemented the KiVa program. The latter was calculated as the difference between the year they had originally registered as program users and each measurement year (the year in which the student responses were provided). The range of responses was 0–7 (0 = less than full academic year).

## Analysis Plan

Mean scores were calculated to examine how often targeted interventions failed, and intraclass correlations (ICC) were calculated to examine the extent to which intervention failures were due to differences between students or differences between schools. Both mean scores and ICCs were calculated separately for victim-perceived and bully-perceived intervention failures.

To investigate factors associated with intervention failure, a series of two-level logistic regression analyses were conducted to predict the within-level probability that the victimization (models 1a and 1b) and bullying perpetration (models 2a and 2b) had continued (versus decreased or stopped) after adult intervention. A two-level regression was chosen to take into account the nested structure of the data (time points, or cases nested within schools—there were several cases from each school in different years). In the models examining intervention failure from victimized students’ perspective, only responses from students who had reported that they had been summoned to a discussion with an adult because they had been victimized were included, and in the models examining failure from bullying perpetrators’ perspective, only responses from students who had reported that they had been summoned to a such discussion because they had been bullying were included. All independent variables were at the within-level and the between-level variance was controlled for. The number of years of KiVa implementation and gender of the student were included in the analyses as control variables. In the first step of each model, after entering the control variables, we tested the effects of grade level and frequency of bullying and victimization separately for victim-perceived (model 1a) and bully-perceived intervention failure (model 2a). In the second step of each model, three additional variables were added. The added variables were outcome-specific and thus different in the two models. The variables used only for victim-perceived intervention failure (model 1b) were duration of victimization, presence of online victimization, and having friends in class. The variables used only for bully-perceived intervention failure (model 2b) were students’ antibullying attitudes and student perceptions of teachers’ and parents’ antibullying attitudes. The analyses were conducted using *M*plus 8.3 (Muthén and Muthén 1998-2023) and the robust version of maximum likelihood estimation (MLR). Missing data was handled using full information maximum likelihood estimation (FIML).

## Results

### Descriptive Statistics

The correlations and descriptive statistics of the study variables are presented in Table 1. Intervention failure—whether from the victimized students’ or the bullying perpetrators’ perspective—was positively correlated with gender (being a boy), grade level, frequency of victimization, and frequency of bullying and negatively correlated with the number of years the school had implemented KiVa. Victim-perceived intervention failure was also positively correlated with the duration of victimization and the presence of online victimization and negatively correlated with having friends in class. Furthermore, bully-perceived intervention failure was negatively correlated with students’ antibullying attitudes and students’ perception of their teachers’ and parents’ antibullying attitudes.

**Table 1:** Within-school correlations and descriptive statistics of the study variables

Variable	1	2	3	4	5	6	7	8	9	10	11	12	*M*	*SD*
1. Intervention failure	-	− .05*	.02*	.26*	.20*	.13*				− .38*	− .34*	− .32*	0.21	0.41
2. Years in KiVa	− .03*	-	.02*	− .06*	− .06*	− .02*				.07*	.07*	.07*	3.43	1.73
3. Boy	.04*	− .03*	-	− .01	.04*	− .03*				− .13*	− .00	− .06*	0.73	0.44
4. Grade level	.13*	− .08*	.04*	-	.03*	.00				− .27*	− .31*	− .24*	6.26	1.65
5. Frequency of bullying	.13*	− .08*	.14*	.08*	-	.34*				− .33*	− .20*	− .22*	1.00	1.14
6. Frequency of victimization	.25*	.01	− .03*	− .07*	.23*	-				− .15*	− .19*	− .13*	0.54	1.01
7. Duration of victimization	.25*	.01^a^	− .01	.18*	.11*	.31*	-							
8. Presence of online victimization	.13*	− .00	− .09*	.02*	.17*	.25*	.14*	-						
9. Friends in classroom	− .24*	.00	− .07*	− .26*	− .12*	− .20*	− .20*	− .05*	-					
10. Students’ antibullying attitudes										-	.43*	.48*	2.71	0.89
11. Perception of teachers’ antibullying attitudes											-	.44*	2.91	1.30
12. Perception of parents’ antibullying attitudes												-	2.96	1.11
*M*	0.27	3.56	0.52	5.96	0.47	1.25	2.56	0.26	2.99					
*SD*	0.45	1.78	0.50	1.66	0.85	1.34	1.52	0.44	1.14					

Correlations and descriptive statistics for victimized students (*n* = 57,835) are below the diagonal. Correlations and descriptive statistics for bullying students (*n* = 44,918) are above the diagonal. Correlations coefficients between binary variables are phi coefficients

*p < .001

^a^*p* < .05

### Prevalence of Failure and Differences Between Schools vs. Students

The mean of victim-perceived intervention failure was 0.27 (0 = victimization decreased or stopped, 1 = the situation did not change, or victimization increased) and the mean of bully-perceived intervention failure was 0.21 (0 = bullying decreased, or stopped, 1 = the situation did not change, or bullying increased). This means that, in most cases, students who had been victimized (73%) and those who had bullied others (79%) reported that the situation had improved after adult intervention.

The intraclass correlations for victim-perceived and bully-perceived intervention failure (ICC = 0.03 and 0.12) indicated that only 3% of the variance in intervention failures reported by victimized students and 12% of the variance in intervention failures reported by bullying perpetrators was due to differences between schools. This means that most of the variance was between students.

### Victim-Perceived Intervention Failure

In model 1a, the dependent variable was victim-perceived intervention failure (victimization had stayed the same or increased versus it had decreased or stopped; see Table 2). Altogether, 11.9% of the within-school variance in victim-perceived intervention failure was explained by the model. The number of years the school had implemented KiVa was associated with decreases in the odds that intervention would fail (OR = 0.99, *p* = 0.024). This means that each additional implementation year decreased the odds by 1%. Grade level was associated with increases in the odds that the intervention would fail (OR = 1.22, *p* < 0.001). This means that each grade level increased the odds of failure by 22%. The likelihood that the intervention would fail was higher for victimized boys (OR = 1.22, *p* < 0.001), when the victimization was more frequent (OR = 1.50, *p* < 0.001) and when the victimized students also bullied others (OR = 1.15, *p* < 0.001).

**Table 2:** Multilevel logistic regression: factors associated with intervention failure

Variable	Victimized students						Bullying students					
	*b*	*SE*	*OR*	95% *CI*	*p*	*Cohen’s d*	*b*	*SE*	*OR*	95%* CI*	*p*	*Cohen’s d*
	Model 1a						Model 2a					
Time (years in KiVa)	− 0.01	0.01	0.99	[0.98–1.00]	.024	− 0.01	− 0.04	0.01	0.97	[0.95–0.98]	.000	− 0.02
Boy	0.20	0.02	1.22	[1.17–1.27]	.000	0.11	0.12	0.03	1.13	[1.07–1.20]	.000	0.07
Grade level	0.20	0.01	1.22	[1.21–1.24]	.000	0.11	0.40	0.01	1.50	[1.47–1.52]	.000	0.22
Frequency of bullying	0.14	0.01	1.15	[1.13–1.18]	.000	0.08	0.33	0.01	1.40	[1.37–1.43]	.000	0.18
Frequency of victimization	0.40	0.01	1.50	[1.47–1.52]	.000	0.22	0.15	0.01	1.16	[1.13–1.19]	.000	0.08
*R*^2^ within	.119	0.00			.000		.172	0.01			.000	
	Model 1b						Model 2b					
Time (years in KiVa)	− 0.02	0.01	0.98	[0.97–0.99]	.000	− 0.01	− 0.01	0.01	0.99	[0.98–1.01]	.496	− 0.00
Boy	0.19	0.02	1.21	[1.16–1.26]	.000	0.11	− 0.07	0.03	0.93	[0.87–0.99]	.015	− 0.04
Grade level	0.11	0.01	1.12	[1.10–1.14]	.000	0.06	0.25	0.01	1.28	[1.26–1.31]	.000	0.14
Frequency of bullying	0.11	0.01	1.11	[1.08–1.14]	.000	0.06	0.14	0.01	1.15	[1.12–1.17]	.000	0.08
Frequency of victimization	0.28	0.01	1.33	[1.30–1.35]	.000	0.16	0.08	0.02	1.09	[1.06–1.12]	.000	0.05
Duration of victimization	0.24	0.01	1.27	[1.24–1.30]	.000	0.13						
Presence of online victimization	0.27	0.04	1.31	[1.22–1.41]	.000	0.15						
Friends in classroom	− 0.29	0.01	0.75	[0.73–0.77]	.000	− 0.16						
Students’ antibullying attitudes							− 0.62	0.02	0.54	[0.51–0.56]	.000	− 0.34
Perception of teachers’ antibullying attitudes							− 0.26	0.01	0.77	[0.75–0.79]	.000	− 0.15
Perception of parents’ antibullying attitudes							− 0.23	0.01	0.79	[0.77–0.81]	.000	− 0.13
*R*^2^ within	.183	0.01			.000		.302	0.01			.000	

*N* for models 1a and 1b = 57,835 (within) and 1990 (between). *N* for models 2a and 2b = 44,918 (within) and 1943 (between)

In model 1b, duration of victimization, online victimization, and whether the victimized student felt that they had friends in the classroom were added to the predictors of model 1a (Table 2). Together, these variables explained an additional 6.4% of the within-school variance in the outcome variable. The likelihood that the intervention would fail was lower for students who felt they had more friends in classroom (OR = 0.75, *p* < 0.001) and higher when the victimization had lasted longer (OR = 1.27, *p* < 0.001) and when the student was also victimized online (OR = 1.31, *p* < 0.001).

### Bully-Perceived Intervention Failure

In model 2a, the dependent variable was bully-perceived intervention failure (Table 2). The within-school variance in bully-perceived intervention failure explained by the model was 17.2%. The number of years the school had implemented KiVa was associated with decreases in the odds that the intervention would fail (OR = 0.97, *p* < 0.001). The likelihood that the intervention would fail was higher for boys than for girls (OR = 1.13, *p* < 0.001). Grade level was associated with increased odds that the intervention would fail (OR = 1.50, *p* < 0.001). Also, the more frequent the bullying was (OR = 1.40, *p* < 0.001) and when the perpetrator was also victimized (OR = 1.16, *p* < 0.001), the higher the chances that the intervention would fail.

In model 2b, students’ antibullying attitudes and their perceptions of teachers’ and parents’ antibullying attitudes were added to the predictors of model 2a (Table 2). The intervention was less likely to fail the stronger the students’ own antibullying attitudes were (OR = 0.54, *p* < 0.001), the stronger they perceived their teachers antibullying attitudes to be (OR = 0.77, *p* < 0.001), and the stronger they perceived their parents’ antibullying attitudes to be (OR = 0.79, *p* < 0.001).

## Discussion

Teachers are not always aware of the bullying taking place in their classroom or school (Haataja et al., 2016), and when they are, they intervene in only about 70–82% of incidents (e.g., Smith & Shu, 2000; Wachs et al., 2019). When teachers or other school personnel do intervene, as many as 20–50% of interventions fail to stop bullying (e.g., Garandeau et al., 2014b; Rigby, 2014). In the current study, we focused on such intervention failures. We examined, first, how often targeted interventions conducted by adults failed using reports from both victimized and bullying students, second, the extent to which intervention failures were due to differences between individual students vs. schools, and third, which factors were associated with failure. We utilized data from approximately 100,000 students from 2032 Finnish schools implementing the KiVa antibullying program, followed for a period of 6 years. The findings increase our understanding of the factors associated with the persistence of bullying despite adult efforts to put an end to it.

Regarding the prevalence of failure, 27% of the victimized students reported that they were still victimized, and 21% of the bullying perpetrators reported that they continued bullying after the adult intervention. By examining intervention failure at the student level (victimized students and bullying perpetrators), these findings expand the results of the study by Johander et al. (2021) who examined the school-level effectiveness of different intervention approaches used in schools implementing the KiVa antibullying program. Overall, these findings indicate that the targeted interventions conducted by adults were quite effective in reducing victimization and bullying in the long term. However, in approximately one out of four cases in which an adult intervened, the intervention failed. Thus, in addition to improving the ways to intervene in victimization and bullying, other actions (e.g., stricter monitoring, referral to a school psychologist) may be necessary when targeted interventions fail.

Only a small portion of the variance (3–12%) in intervention failures was due to differences between schools. This means that when intervening in bullying, it is important to focus on the differences between the students involved in the bullying case. In the current study, we did exactly this by examining factors associated with the intervention failure at the individual level. Although effect sizes were small, all the effects were consistent with our expectations. The likelihood that the intervention would fail increased as the students got older. This finding is consistent with the results of Johander et al. (2021) who found that targeted interventions were less effective in secondary schools compared to primary schools, as well as meta-analyses indicating that whole-school antibullying programs tend to work better among younger students (e.g., Hensums et al., 2022; Yeager et al., 2015). According to the present findings, however, it is not only a matter of primary schools having a greater capacity than secondary schools for dealing with bullying, but of developmental differences as well; every additional year made the intervention failure more likely. In addition to the finding that bullying seems to be more selective in secondary school than in primary school with more bullies targeting fewer victims (Kärnä et al., 2011a), this shows that the plight of victimized students may be especially difficult in higher grades. To reduce the prevalence of bullying in general, systematic antibullying work needs to be reinforced in secondary schools.

Both the frequency of victimization and the frequency of bullying positively predicted intervention failure. In line with a previous study, the duration of victimization also positively predicted the intervention failure (Garandeau et al., 2014b). Together, these results suggest that the more intense the victimization and bullying is, i.e. the more often it occurs and the longer it has lasted, the harder it is to intervene in it. These results highlight the importance of intervening in bullying as early as possible to prevent it from becoming chronic and turning into a fixed relational pattern that is hard to break. Also, since more frequent victimization can be an indication of being targeted by more than one perpetrator (Rigby, 2020), it is important to ensure that all the students involved are confronted. In cases of more frequent bullying perpetration, other possible co-occurring problems (e.g., aggressive-impulsive behavior and retaliatory attitudes; O’Brennan et al., 2009) need to be addressed.

The victimized students’ own bullying behavior as well as the bullying perpetrators’ own victimization both increased the likelihood that the intervention would fail. This means that intervening in cases where the students involved are both victimized and also bully others (i.e., bully-victims) was more difficult. Bully-victims are known to be more maladjusted than either “pure” victims or “pure” bullies. They tend to be the most rejected (e.g., Veenstra et al., 2005) and experience more internalizing (e.g., anxiety, depression; Cook et al., 2010), externalizing (e.g., conduct and hyperactivity symptoms; Kelly et al., 2015), and school adjustment problems (e.g., lack sense of bonding to school, experience less teacher support; Berkowitz & Benbenishty, 2012; Juvonen et al., 2003). Furthermore, it is possible that bully-victims feel entitled to bully others due to their own victimization and other students feel entitled to bully them due to their aggression. This all might make intervening successfully harder. Interventions were also more likely to fail in cases where the targeted student had been victimized online and had fewer friends in the class. The co-occurrence of bullying and victimization, exposure to several forms of bullying, and the absence of social support all hinder the effectiveness of adult interventions and therefore should be taken into consideration as part of intervention efforts. Especially when some of the bullying has been taking place online, teachers may not be aware of it and may lack the skills or the means to successfully intervene. Thus, more attention should be paid to this in teacher training and in antibullying programs.

The perpetrators’ own antibullying attitudes and their perception of teachers’ and parents’ antibullying attitudes were all negatively associated with the intervention failure, which is in line with the theory of planned behavior (Ajzen, 1991). The stronger the perpetrators’ own antibullying attitudes were and the stronger they thought their teachers’ and parents’ antibullying attitudes were, the more likely they were to stop bullying after the intervention. Although not directly examined in the current study, it could be hypothesized that teachers’ and parents’ attitudes affect students’ own attitudes, which in turn affect the likelihood of intervention success. As has also been shown in previous research, students observe their teachers’ attitudes and modify their behavior accordingly (Saarento et al., 2015). The current results suggest that they do the same with regard to the attitudes of their parents. Thus, teachers and parents should be more aware of their influence as a role model and how easily their attitudes are transferred and can influence the behavior of children and adolescents.

Regarding the covariates, the number of years schools had implemented the KiVa program was associated with a lower likelihood that the intervention would fail. It is possible that, over the years, the school personnel implementing the program have gained experience and become more skillful in addressing cases of bullying or that the implementation of the program has created an increasingly strong antibullying atmosphere in the school, which results in higher effectiveness of the interventions. With regard to the gender of the students, the likelihood that the intervention would fail was higher for boys. Indeed, boys are more likely than girls to bully others and to be victimized (e.g., Cook et al., 2010). These results suggest that the victimization and bullying perpetration of boys might be also harder to tackle. Although boys are not necessarily less responsive to antibullying programs as a whole (Gradinger et al., 2015), research has shown that, compared to girls, boys tend to experience more distant and conflictual relationships with teachers (Koepke & Harkins, 2008). This could explain the lower compliance of male bullying perpetrators with teachers’ requests to stop bullying. This is in line with the results of Johander et al. (2022), who also found that boys were less likely to say that they would stop their bullying behavior as a response to an intervention. This lower responsiveness of male perpetrators to the interventions could also be the reason why victimization was more likely to continue for male victims. Indeed, boys are more likely to be victimized by other boys than by girls (Sainio, 2013).

### Limitations

The key strengths of this study lie in its large sample size, the examination of both victimized students and bullying perpetrators perspectives, and the consideration of a wide range of factors predicting failure of targeted interventions. It also has a number of limitations. To preserve the anonymity of the responses, the students who participated in the study logged in to the survey with a school-level ID. Thus, it is unknown whether some of the victimized or bullying students who reported that their situation was addressed by the school personnel had participated in the discussions only once or several times within a school year or during the years of the study. It is also unknown whether some of the students were involved in the discussions because they had been victimized one year and because they had been bullying others another year. If individual students could be followed from one year to the next, the data could be analyzed as a three-level model (targeted interventions nested within students nested within schools), which would increase the validity of the study.

Moreover, we did not observe how the interventions were conducted. Thus, we do not know what exactly was said in the discussions between the adults and the students involved. Furthermore, this study examined how different factors were associated with intervention failure. However, it is unclear whether the intervention failure was an antecedent, rather than a consequence, of some of the examined factors. For instance, it is possible that the victimization lasted longer because the intervention failed in the first place rather than duration of victimization making interventions failures more likely.

Finally, the study relied on retrospective student reports rather than (for instance) observations of whether the interventions had been successful. An obvious limitation is therefore memory bias. Moreover, some interventions (and their consequences) might be more memorable than others, and therefore, the memories of these events may be more likely to be retrieved when responding to the survey. Bullying students’ reports might also be affected by social desirability bias; perhaps, they are more likely to report that they responded to the intervention in a way that was expected of them, and therefore, reports of success may have been overestimated in bullying perpetrators’ responses. The difference in victim-reported and bully-reported intervention failures (although not huge) suggests that this might be the case. Hence, it is important to use both informants when examining the effectiveness of targeted interventions.

### Implications and Future Research Directions

The targeted antibullying interventions conducted by adults in KiVa schools were overall quite effective in reducing victimization and bullying in the long term. However, approximately in one out of four cases where an adult intervened, the intervention failed. Most of the variation in intervention failures was between students. Thus, in order to better understand the challenges of antibullying interventions and to identify actions needed in the most challenging cases, it is important to consider the individual characteristics of the students involved. This study suggests that such challenges could be related to the pro-bullying attitudes of the perpetrators, or the lack of friends or aggressiveness of the victimized students. Also, the finding that presence of online victimization positively predicted intervention failure suggests that teachers need to be trained to better recognize and address online bullying.

This study focused on how some characteristics of the victimized students or bullying perpetrators are associated with intervention failure. However, other factors might have an effect on whether the intervention will be successful and were not taken into consideration. For example, the level of perceived popularity of the bullying perpetrators, which can be an indicator of how socially rewarding bullying is for them, has been shown to be associated with reduced effectiveness of whole antibullying programs for bullying perpetration (Garandeau et al., 2014a) and might also make perpetrators more resistant to targeted interventions. Moreover, more contextual factors should be considered in future research. These include the quality of teacher-student relationships, the level of collaboration between school personnel and parents, and the school social climate and the support provided by headmasters for antibullying work (Ahtola et al., 2013), which might matter for the success of the interventions.

We believe that it is important for the field to shift its focus from the examination of intervention success to the examination of intervention failure. When bullying stops after an adult intervention, it is impossible to determine with certainty whether it stopped due to the intervention or whether it would have stopped anyway. In cases where the bullying continued after the intervention, there is clear evidence that the intervention failed and it is important to study why. We encourage future studies to identify more factors associated with intervention failure, such as students’ psychological reactance and callous-unemotional traits, in order to improve our knowledge of the key obstacles to successful interventions and guide the development of more effective antibullying strategies.

## Data Availability

Fully anonymized data and analysis code are available from the first author upon reasonable request.
